# Advances in the Chemical and Biological Characterization of Amaryllidaceae Alkaloids and Natural Analogues Isolated in the Last Decade

**DOI:** 10.3390/molecules25235621

**Published:** 2020-11-29

**Authors:** Marco Masi, Roberta Di Lecce, Alessio Cimmino, Antonio Evidente

**Affiliations:** Dipartimento di Scienze Chimiche, Università di Napoli Federico II, Complesso Universitario Monte Sant’Angelo, Via Cintia 4, 80126 Napoli, Italy; marco.masi@unina.it (M.M.); roberta.dilecce@unina.it (R.D.L.); alessio.cimmino@unina.it (A.C.)

**Keywords:** Amaryllidaceae, alkaloids, natural analogues, last decade

## Abstract

Amaryllidaceae are bulbous wild and cultivated plants well known for their beautiful flowers and pharmaceutical applications, essentially due to the alkaloids and flavonoids content. Hundreds of alkaloids have been isolated until now and several scientific publications reported their sources, chemical structures, and biological activities. During the last decade, some unstudied Amaryllidaceae plants were the object of in-depth investigations to isolate and chemically and biologically characterize new and already known alkaloids as well as some analogues. This review describes the isolation and chemical and biological characterization of the Amaryllidaceae alkaloids, and their analogues obtained in the last decade, focusing the discussion on the new ones.

## 1. Introduction

The Amaryllidaceae are wild [1] and cultivated plants in several countries. They are considered ornamental plants for their beautiful flowers and to produce volatile oils. They are dominant plants in Andean South America, the Mediterranean basin, and southern Africa [2,3]. The main metabolites synthesized by Amaryllidaceae are essentially alkaloids which accumulate in their bulbs.

Several transcriptomic and biochemical studies described the molecular features involved in the biosynthesis of Amaryllidaceae alkaloids, including enzymes from the shikimate and phenylpropanoid pathways, were recently reviewed [4]. Amaryllidaceae plants consist of ca. 85 genera and 1100 species, and ca. 600 structurally diverse alkaloids have been isolated from plants and grouped in 12 ring-types [5,6].

The investigation on the Amaryllidaceae alkaloids began in 1877 with the isolation of lycorine from *Narcissus pseudonarcissus* [7] and then the interest around this group of naturally occurring compounds increased because of the large spectrum of their biological activities. These include antitumor, antibacterial, antifungal, antimalarial, antiviral, analgesic, and cholinesterase (AChE and BuChE) inhibitory activities. The uniqueness of these alkaloid structures provided a viable platform for phytochemical-based drug discovery [6,8,9]. Galanthamine represents the main medicinal application of Amaryllidaceae alkaloids and is commercialized as an Alzheimer’s drug [2].

Detailed investigations were carried out on the in vitro antiproliferative, apoptosis-inducing, and antiinvasive activities of Amaryllidaceae alkaloids and their derivatives, aiming to analyze their potential anticancer activity. These studies showed the potency of several Amaryllidaceae alkaloids as well as related isocarbostyryls (pancratistatine and narciclasine) against some solid tumors, reporting the mode of action to explain their cytotoxic activity [8,9,10,11,12,13,14,15,16,17].

Some Amaryllidaceae alkaloids also exhibited good growth inhibitory activities against several fungal pathogens and this activity was further investigated due to the emergence of drug-resistant strains and the loss of efficacy of existing antifungals [18]. Similarly, the antimalarial effect of some crinane alkaloids was investigated against various strains of the parasite *Plasmodium falciparum* and these studies afforded useful information on the anti-plasmodial pharmacophore [19].

Amaryllidaceae plants are also known to be poisonous and these toxic effects have to be taken into account [20].

Recently, the assignment of the absolute configuration to these alkaloids, which is closely related to their biological activity, was also reviewed [21]. Finally, the synthesis, the chemodiversity, chemotaxonomy, and chemoecology of some Amaryllidaceae alkaloids was reported [22].

This review reports the advances in the chemical and biological characterization of Amaryllidaceae alkaloids and natural analogues isolated in the last decade, focusing on the new ones.

## 2. Novel Alkaloids from Different Unstudied Amaryllidaceae Plants

### 2.1. Alkaloids from Phaedranassa dubia

**Figure 1:**
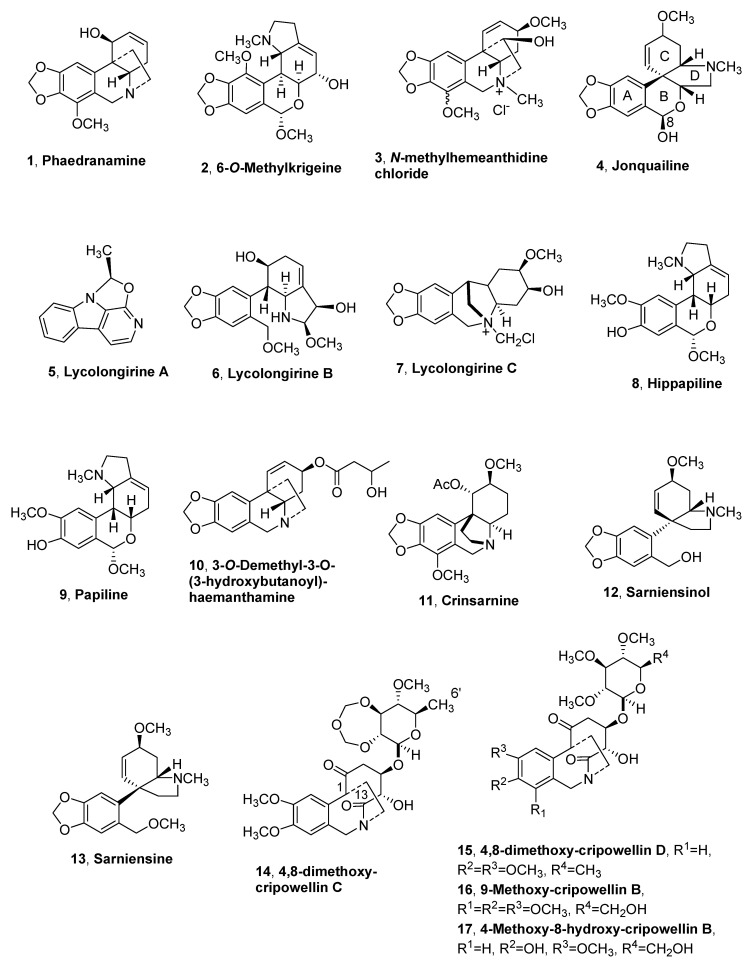
Alkaloids and natural analogues isolated from *Phaedranassa dubia*, *Nerine huttoniae*, *Zephyranthes candida*, *Narcissus jonquilla quail*, *Lycoris longituba*, *Hippeastrum papilio*, *Nerine sarniensis*, and *Crinum latifolium*.

*Phaedranassa dubia* belong to *Phaedranassa* [23], which is a small genus in the Amaryllidaceae family comprising eleven species: eight endemic to Ecuador, three known from Colombia, and one from Costa Rica [24]. From the bulbs of *P. dubia*, collected in Colombia, a new alkaloid, named phaedranamine (**1**, Figure 1, Table 1), and belonging to the crinine-type, was isolated together with the well-known Amaryllidaceae alkaloids *epi*-*nor*-galanthamine, galanthamine, haemanthamine, pseudolycorine, sanguinine, ungeremine, and zefbetaine [25].

All the alkaloids isolated were tested against parasitic protozoa and for cytotoxicity. Ungeremine, pseudolycorine, and haemanthamine showed good activity in vitro against *Trypanosoma brucei rhodesiense*, *Trypanosoma cruzi,* and *Plasmodium falciparum*, with IC_50_ (half maximal inhibitory concentration) values in the range of 3.66 mM or lower. Ungeremine showed higher toxicity than the other alkaloids but was inactive against *Leishmania donovani* and showed cytotoxic activity against L6 cells (rat skeletal myoblasts, IC_50_ = 65.28 mM). Related compounds with quaternary nitrogen showed strong antiprotozoa activity and the anti-plasmodial activity exhibited by ungeremine, pseudolycorine, and haemanthamine does not depend on interactions with heme [25]. These results increased the knowledge on the structure-antiprotozoal activity relationships in Amaryllidaceae alkaloids, which are scantly investigated. Previously, only the role of a methylenedioxy group and a tertiary non-methylated nitrogen was reported to impart higher activity [38].

### 2.2. Alkaloid from Nerine huttoniae

*Nerine huttoniae* is a species belonging to the *Nerine* genus [23], which comprises ca. 23 perennial bulbous species native to southern Africa. This Amaryllidacea is a summer growing, evergreen species, and was essentially found in the western part of the Eastern Cape Province of South Africa [39,40]. It was used in folk medicine and in particular by Sotho and Zulu tribes [41].

From the bulbs of *N. huttoniae,* a new alkaloid, belonging to the homolycorine-type of Amaryllidaceae alkaloids and named 6-*O*-methylkrigeine (**2**, Figure 1, Table 1), was isolated together with the known oxokrigenamine. Compound **2** did not exhibit acetylcholine esterase inhibitory activity when tested at a concentration of 50 μg mL^−1^ [26].

### 2.3. Alkaloids from Zephyranthes candida

*Zephyranthes candida* is an Amaryllidacea plant well-known for its use in folk medicine and in particular in China [42]. In fact, its organic extract showed potent cytotoxicity against tumor cells. Fifteen alkaloids were isolated from the whole plant extract and identified as *N*-methylhemeanthidine chloride, *N*-methyl-5,6-dihydroplicane, *O*-methylnerinine, *N*-ethoxycarbonylethylcrinasiadine, *N*-ethoxycarbonylpropylcrinasiadine, *N*-phenethylcrinasiadine *N*-isopentylcrinasiadine, hemeanthamine, 3-*epi*-macronine, (+)-tazettine, *N*-methylcrinasiadine, trisphaeridine, 5,6-dihydrobicolorine, lycorine, and nigragillin. When tested for their cytotoxicity against five human cancer cell lines and the Beas-2B immortalized (noncancerous) human bronchial epithelial cell line, the *N*-methyl-5, 6-dihydroplicane, hemeanthamine, *N*-isopentylcrinasiadine, and lycorine showed toxicity, with IC_50_ values ranging from 0.81 to 13 μM [42].

Subsequently, the new *N*-methylhemeanthidine chloride (**3**, Figure 1, Table 1 and Table 2) was isolated from *Z. candida* and exhibited potent cytotoxicity on a spectrum of tumor cells.

In particular, the cytotoxic activity of compound **3** was deeply investigated using multiple cell lines derived from human pancreatic cancer, which is one of the most mortal and refractory human malignancies. Compound **3** showed a very strong cytotoxic activity on cancerous cells but was not toxic to healthy ones. Although the mode of action of compound **3** remains un-determined, the results obtained comparing its cytotoxicity with the activity of the chemotherapeutic agent gemcitabine allowed the proposal of compound **3** as a promising drug against pancreatic cancer [27]. Further studies were carried out on the anticancer activity of compound **3**, suggesting that this haemanthidine derivative has a tumor suppressive role of NOTCH (Notch Pathway Modulators as Anticancer Chemotherapeutics), signaling in acute myeloid leukemia (AML). The reactivation of this mechanism in a new attractive opportunity to develop an alternative therapy against AML [28].

### 2.4. Alkaloids from Narcissus jonquilla quail

*Narcissus jonquilla quail*, native to Spain and Portugal, has now become naturalized in many regions of Europe and the United States. The extract of bulbs collected in Middlesex county of southeast England, as shown by GC analysis, showed the presence of galanthamine and haemanthamine as the main alkaloids [44].

Further investigation carried out by Masi et al. [29] allowed to isolate abundant amounts of haemanthamine, lycorine and narciclasine, and a new alkaloid, named jonquailine (**4**, Figure 1, Table 1 and Table 2) and belonging to the pretazettine group of Amaryllidaceae alkaloids [29]. An extensive work was carried out by comparing the ECD spectrum of jonquailine and tazettine, and the ECD data reported in the literature for pretazettine showed that compound **4** and pretazettine have the same absolute configuration at the B/C and B/D ring junctions, while they are empimers at C-8. The stereochemistry at C-8 was not previously assigned in pretazettine and thus is not assigned in the alkaloid **4** [44]. Subsequently, the absolute configuration, *R,* at C-8 of compound **4** was assigned by density functional theory (DFT) calculations of chiroptical properties, namely electronic circular dichroism (ECD), vibrational circular dichroism (VCD), and optical rotatory dispersion (ORD). These results confirmed the absolute configuration of jonquailine and allowed the assignment of an *S* configuration to C-8 of pretazettine [45].

Jonquailine showed anticancer activity against drug-resistant human tumor models with diverse mechanisms and displayed synergy with paclitaxel. These results and literature data demonstrated that the hydroxylation at C-8 is an important feature to impart the anticancer activity, which is independent from its stereochemistry as both jonquiline and pretazettine showed significant activity, while tazettine, lacking this hydroxyl group, had no activity [29].

### 2.5. Alkaloids from Lycoris longituba

*Lycoris longituba* is native in Jiangsu province in China [46], and its bulbs were used in folk medicine for different skin diseases [47].

Three novel alkaloids, named lycolongirine A, lycolongirine B, and lycolongirine C (**5**–**7**, Figure 1, Table 1 and Table 2), were isolated from the bulbs of *L. longituba* collected in Baohua Mountain, Jiangsu province. Twenty-two already known alkaloids were also obtained [30]. Lycolongirines B and C (**6** and **7**) belong to the ismine and to montanine-type alkaloids, respectively. The known alkaloids were identified as: incartine, norharmane, harmane, perlolyrine, lycorine, hippamine, *N*-chloromethyl narcissidine, trisphaeridine, *N*-methylcrinasiadine, (+)-haemanthidine, (-)-haemanthidine, galanthamine, *N*-norgalanthamine, *N*-chloromethyl galanthamine, 11β-hydroxy galanthamine, sanguinine, *N*-chloromethyl lycoramine, *O*-demethyllycoramine, and tazettine deoxytazettine. All the isolated alkaloids showed different degrees of neuroprotective activities against CoCl_2_-, H_2_O_2_-, and Aβ_25–35_-induced SH-SY5Y cell injuries, while *N*-methylcrinasiadine galanthamine *N*-norgalanthamine, *N*-chloromethylgalanthamine, 11β-hydroxygalanthamine, sanguinine, *N*-chloromethyl lycoramine, *O*-demethyllycoramine, and deoxytazettine strongly hinibited Acetylcholinesterase (AChE) activities [30]. 

### 2.6. Alkaloids from Hippeastrum papilio

*Hippeastrum papilio* is an Amarylidacea collected in Brazil, and from its dried bulbs three novel alkaloids were isolated, named hippapiline, papiline, and 3-*O*-demethyl-3-*O*-(3-hydroxybutanoyl)-haemanthamine (**8**–**10**, Figure 1, Table 1). Alkaloids **8**–**10** belong to homolycorine-, ismine-, and crinine-type Amaryllidaceae alkaloids. Also, six already known alkaloids were isolated from the same plant and identified as haemanthamine, galanthamine, narwedine, 11b-hydroxygalanthamine, apogalanthamine, and 9-*O*-demethyllycosinine B [31].

### 2.7. Alkaloids from Nerine sarniensis

*Nerine sarniensis* belonging to a genus well-known as ornamental plants is an herbaceous bulbous perennial species. *Nerine* genus is comprised of 24 species in the Amaryllidaceae family and is endemic to South Africa and a few neighboring countries [2]. *N. sarniensis* is restricted to the Western Cape of South Africa [48].

From the organic extract of the bulbs of *N. sarniensis,* a new crinine-type alkaloid named crisarnine (**11**, Figure 1, Table 1 and Table 2), and two new mesembrine-type alkaloids named sarniensinol (**12**, Figure 1, Table 1) and sarniensine (**13**, Figure 1, Table 1 andTable 2), were isolated. Also, several known alkaloids were isolated and identified as tazettine, lycorine, and 3-*epi*-macronine, as the main alkaloid, and bowdensine, sarniensine, hippadine, and 1-*O*-acetyl-lycorine [32,33].

The extract of *N. sarnine* bulbs showed strong larvicidal activity with an LC_50_ value of 0.008 μg μL^−1^ against first instar *Aedes aegypti* larvae, and with an LD_50_ value 4.6 μg/mosquito against adult female *Ae. aegypti*, which is the major vector of dengue and yellow fevers and the Zika virus. All the alkaloids were tested against *Ae. aegypti* and only crinsarnine (**12**) showed adulticidal activity with an LD_50_ = 2.29 – 0.049 ± μg/mosquito [48], while sarniensine (**10**), at a concentration of 0.1 μg μL^−1^, exhibited strong adulticidal activity, with an LD_50_ value of 1.38 ± 0.056 μg/mosquito [33].

### 2.8. Alkaloids from Crinum latifolium 

*Crinum latifolium* is widespread in the upper Gangetic Plain. It is also cultivated and used in folk medicine [16]. This Amaryllidacea belongs to *Crinum* L., which is the only genus mainly distributed in Africa, America, Australia, and southern Asia [49,50,51,52]. This genus contains ca. 110 species [53].

Four new bioactive alkaloids, belonging to the crynine-type subgroup and named 4,8-dimethoxy-cripowellin C, 4,8-dimethoxycripowellin D, 9-methoxy-cripowellin B, and 4-methoxy-8-hydroxy-cripowellin B (**14**–**17**, Figure 1, Table 1 andTable 2), were isolated from the *C. latifolium* bulbs extract, together, the known cripowellin [34]. The alkaloids **14**–**17** and cripowellin showed strong cytotoxicity against seven lung cancer cell lines with IC_50_ < 30 nM. Compounds **16** and **17** also showed significant antimicrobial activity with IC_50_ values < 0.50 mM when tested against the Gram+ bacteria, such as *Streptococcus pneumoniae*, *Staphylococcus aureus,* and *Staphylococcus epidermidis*, and Gram– bacteria such as *Klebsiella pneumoniae*, *Pseudomonas aeruginosa*, *Haemophilus influenzae*, *Enterobacter cloacae*, and *Shigella dysenteriae*. All the alkaloids also showed antioxidant activity in the ABTS+ and DPPH tests. In addition, alkaloids **14**–**17** and cripowellin, tested in vitro for their anti-inflammatory potential, showed comparable inhibition of Cox(Cyclo-oxigenase)-1 (>64%) with positive control SC-560 and of Cox-2 (>90%) with positive control NS–398, respectively [34]. These results suggested that the cleavage between C-1 and C-13 in crinane alkaloid skeleton is a structural feature important to impart biological activity, in which also the presence of the hydroxyl at C-6′ could play a role [34].

### 2.9. Alkaloids from Zephyranthes grandiflora 

*Zephyranthes grandiflora*, as the above-reported for *Z. candida,* belong to genus *Zephyranthes*, and consists of 60 species which are distributed mainly in the warm-temperate regions of the Western hemisphere [54,55]. These plants are well-known for their ornamental use and medicinal properties [56].

**Figure 2:**
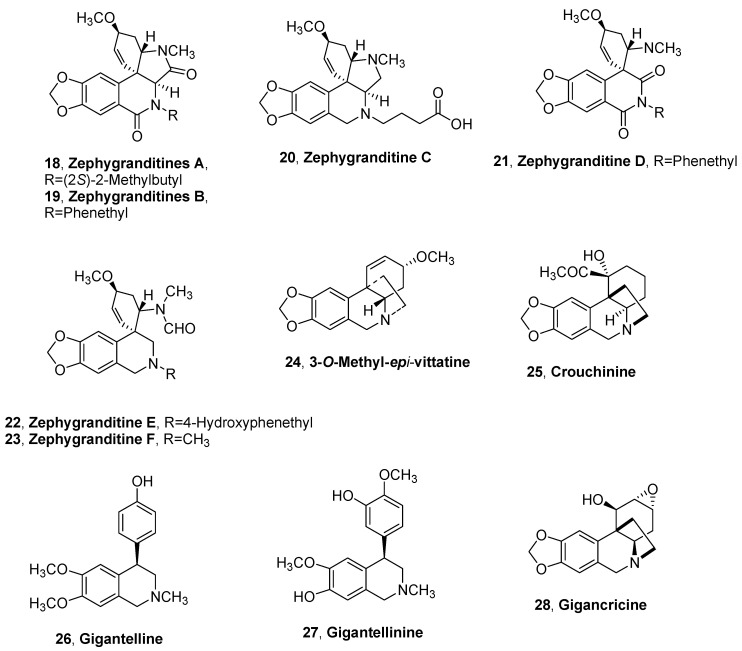
Alkaloids isolated from *Zephyrantes grandiflora*, *Brunsvigia natalensis*, and *Crinum jagus*.

Six new 4a-*epi*-plicamine-type alkaloids, named zephygranditines A–C (**18**–**20**, Figure 2 and Table 1 and Table 2) and zephygranditines D–F (**21**–**23**, Figure 2, Table 1), including three novel 11,12-seco-plicamine-type alkaloids, were isolated from the organic extract of *Z. grandiflora.* Zephygranditines A–C (**18**–**20**) alkaloids showed cytotoxic activity against seven malignant melanoma cell lines with IC_50_ values < 20 μM, while only alkaloids **18** and **19** exhibited anti-inflammatory activity in both assays of inhibitory activity for nitric oxide production and Cox-1/Cox-2 [56].

### 2.10. Alkaloids from Brunsvigia natalensis

*Brunsvigia natalensis*, also named as Natal Candelabra Flower, occurs in South Africa, Lesotho, and Swaziland, and was used in traditional medicine [35].

3-*O*-Methyl-*epi*-vittatine and crouchinine (**24** and **25**, Figure 2, Table 1), two new 5,10b-ethanophenanthridine bridge alkaloids, were isolated from *B. natalensis* together with (2*R*)-7-hydroxyflavan and a novel ceramide, named brunsceramide [57].

### 2.11. Alkaloids from Crinum jagus

*Crinum jagus* (syn. = *Crinum giganteum*) grows in Senegal and belongs to a genus which was shown to be very rich in crinine-type alkaloids [36]. The metabolites present in the aqueous and organic extract of *C. jagus* showed potential for the treatment of inflammatory processes [58], antibacterial [59], sedative [60], inhibition of cholinesterases [61], and antiviral [62] activities. 

Three undescribed Amarylidaceae alkaloids, named gigantelline (**26**, Figure 2, Table 1), gigantellinine (**27**, Figure 2, Table 1 and Table 2) and gigancrinine (**28**, Figure 2, Table 1), and belonging to the cherylline- and crine-type subgroups, were isolated from the acid organic extract of *C. jagus* bulbs. The new alkaloids were extracted together with some already known alkaloids identified as sanguinine, cherylline, lycorine, crinine, flexinine, and the isoquinolinone derivative hippadine [37]. Cherylline, gigantellinine, crinine, flexinine, and sanguinine inhibited the activity of AChE in a dose-dependent manner, and the inhibition by sanguinine was remarkably effective (IC_50_ = 1.83 ± 0.01 μM), while cherylline and hippadine showed weak cytotoxicity at 100 μM [37]. 

## 3. Conclusions

This review described the alkaloids and their natural analogues isolated in the last decade from different Amaryllidaceae species, focusing the discussion on the new ones. The world region in which they grow and their diffused use in folk medicine was also reported. Together with the new alkaloids, some new analogues were reported as well as other well-known alkaloids and other metabolites isolated from Amaryllidaceae plants. The description of isolation and chemical and biological characterization of the new alkaloids follows a chronological order and their biological activities in the tests were described and listed in Table 1, together with the plant source and literature. In some cases, results on the structure activity relationships were also described.

## Figures and Tables

**Table 1:** Amaryllidaceae alkaloids and natural analogues isolated in the last decade.

Alkaloid	Amaryllidacea	Reference
Phaedranamine (**1**, Figure 1)	*Phaedranassa dubia*	[25]
6-*O*-Methylkrigeine (**2**, Figure 1)	*Nerine huttoniae*	[26]
*N*-methylhemeanthidine chloride (**3**, Figure 1)	*Zephyranthes candida*	[27][28]
Jonquailine (**4**, Figure 1)	*Narcissus jonquilla quail*	[29]
Lycolongirine A (**5**, Figure 1)	*Lycoris longituba*	[30]
Lycolongirine B (**6**, Figure 1)	“ ^1^	“
Lycolongirine C (**7**, Figure 1)	“	“
Hippapiline (**8**, Figure 1)	*Hippeastrum papilio*	[31]
Papiline (**9**, Figure 1)	“	“
3-*O*-Demethyl-3-*O*-(3-hydroxybutanoyl)- haemanthamine (**10**, Figure 1)	“	“
Crinsarnine (**11**, Figure 1)	*Nerine sarniensis*	[32]
Sarniensinol (**12**, Figure 1)	“	[33]
Sarniensine (**13**, Figure 1)	“	[32]
4,8-Dimethoxy-cripowellin C, (**14**, Figure 1)	*Crinum latifolium*	[34]
4,8-Dimethoxy-cripowellin D (**15**, Figure 1)	“	“
9-Methoxy-cripowellin B (**16**, Figure 1)	“	“
4-Methoxy-8-hydroxy-cripowellin B (**17**, Figure 1)	“	“
Zephygranditine A (**18**, Figure 2)	*Zephyranthes grandiflora*	[35]
Zephygranditine B (**19**, Figure 2)	“	“
Zephygranditine C (**20**, Figure 2)	“	“
Zephygranditine D (**21**, Figure 2)	“	“
Zephygranditine E (**22**, Figure 2)	“	“
Zephygranditine F (**23**, Figure 2)	“	“
3-*O*-Methyl-*epi*-vittatine (**24**, Figure 2)	*Brunsvigia natalensis*	[36]
Crouchinine (**25**, Figure 2)	“	“
Gigantelline (**26** Figure 2)	*Crinum jagus*	[37]
Gigantellinine (**27** Figure 2)	“	“
Gigancrinine (**28** Figure 2)	“	“

^1^ This menas that the table cell contain the same concept of the previous cell.

**Table 2:** Biological activities of Amaryllidaceae alkaloids and natural analogues isolated in the last decade.

Alkaloid	Biological Activity	Reference
*N*-methylhemeanthidine chloride (**3**, Figure 1)	Cytotoxic Anticancer against AML	[27] [28]
Jonquailine (**4**, Figure 1)	Anticancer activity	[29]
Lycolongirine A (**5**, Figure 1)	Neuroprotective	[30]
Lycolongirine B (**6**, Figure 1)	“ ^1^	“
Lycolongirine C (**7**, Figure 1)	“	“
Crinsarnine (**11**, Figure 1)	Insecticidal	[32]
Sarniensine (**13**, Figure 1)	Insecticidal	[32]
4,8-Dimethoxy-cripowellin C, (**14**, Figure 1)	Cytotoxic Antioxidant Anti-inflammatory	[34,43]
4,8-Dimethoxy-cripowellinD (**15**, Figure 1)	“	“
9-Methoxy-cripowellin B (**16**, Figure 1)	Cytotoxic Antioxidant Anti-inflammatory Antimicrobial	“
4-Methoxy-8-hydroxy-cripowellin B (**17**, Figure 1)	“	“
Zephygranditine A (**18**, Figure 2)	Cytotoxic Anti-inflammatory	[35]
Zephygranditine B (**19**, Figure 2)	“	“
Zephygranditine C (**20**, Figure 2)	Cytotoxic	“
Gigantellinine (**27** Figure 2)	Inhibition of AChE	“

^1^ This menas that the table cell contain the same concept of the previous cell.
